# Recognition and management of hemorrhaging in combination with emerging enterogenic sepsis during a hepatectomy: a case report

**DOI:** 10.1186/s12871-023-02108-x

**Published:** 2023-05-30

**Authors:** Yingjie Chen, Yanling Liao, Xiaoying Chen, Hanliang Fan, Daoyi Lin, Ting Zheng, Xiaohui Chen, Cansheng Gong, Fei Gao, Jundan Jiang, Xiaochun Zheng

**Affiliations:** Department of Anesthesiology, Shengli Clinical Medical College of Fujian Medical University, Fujian Provincial Hospital, Fuzhou, China

**Keywords:** Emerging enterogenic sepsis, Hemorrhage, Hepatectomy, Case report

## Abstract

**Background:**

Patients with hemorrhagic shock may develop emerging enterogenic sepsis due to damage to the intestinal mucosal barrier and translocation of intestinal bacteria and endotoxins caused by ischemic injury. Because of the dual effects of anesthesia state and hemorrhagic shock, perioperative emerging enterogenic sepsis is even more rare and insidious.

**Case presentation:**

We reported a case of 56-year-old man who underwent right hepatectomy for intrahepatic bile duct stones. Severe hemorrhage occurred during the procedure and the hemodynamics neither improved nor worsened after rehydration therapy and vasoactive drug administration. Based on the patient’s history and clinical presentation, a possible enterogenic sepsis was considered. After anti-infective treatment and hormone supplementation, the patient’s circulation improved significantly and he had an uneventful recovery.

**Conclusion:**

The possibility of emerging enterogenic sepsis in hemorrhagic shock must always be taken into consideration. Familiarity with the risk factors and pathophysiological alterations of enterogenic sepsis is a prerequisite for early recognition and sound clinical decision making.

**Supplementary Information:**

The online version contains supplementary material available at 10.1186/s12871-023-02108-x.

## Introduction

Hepatectomies are one of the primary treatment strategies for a wide range of benign and malignant liver diseases, with approximately 7,000 to 10,000 hepatectomies being performed each year in the United States alone [1]. With the continued improvement of surgical techniques and perioperative management, the mortality rate of hepatectomies has decreased significantly over the past 25 years [2]. However, intraoperative hemorrhaging or hemorrhagic shock have remained major risks for patients undergoing hepatectomies. Rapid and massive intraoperative bleeding causes a reduction in effective circulating blood volume and inadequate tissue perfusion. Notably, the intestine has been identified as one of the first organs involved in ischemia-reperfusion injuries. The most serious consequences of ischemic injuries to the gut are damage to the intestinal mucosal barrier and translocation of intestinal bacteria and endotoxins into the bloodstream. At worst, sepsis may develop as a result, thereby exacerbating the development of multiple organ dysfunction syndrome (MODS) [3].

While recognition of the important role that enterogenic sepsis plays in shock has grown in in the medical community over the years, there is often still a general lack of awareness of the speed with which it can develop. As such, there are few reports on the anesthetic management of intraoperative hemorrhages combined with emerging enterogenic sepsis, while studies documenting the function of the intestinal barrier in hemorrhagic shock are equally rare. In this case, we report our experience in performing anesthetic management for an intraoperative hemorrhage combined with emerging enterogenic sepsis.

## Case presentation

Our subject is a 56-year-old male weighing 53 KG and 160 cm tall who had a history of multiple cholelithiasis, as well as multiple choledochotomies over the past 30 years. He was admitted to the hospital complaining of recurrent right upper abdominal pain over the span of one week and abdominal CT showed varying degrees of intrahepatic bile duct dilatation with multiple stones.Preoperatively diagnosed with the following: (1) intrahepatic bile duct stones with cholangitis; and (2) biliary cirrhosis. The patient had been treated with anti-infective therapy at a lower hospital prior to admission, and continued with ertapenem for one week after admission.The treatment plan of an elective right hepatectomy with bile-intestinal anastomosis and reconstruction was decided. The preoperative examination showed approximately normal results, except for a mild increase in transaminases.

The patient entered the operating room at 10:40 on the day of surgery. Considering the patient’s history of multiple biliary surgeries and the likelihood of finding a large number of adhesions in the abdominal cavity, the operation was much more difficult than usual. In addition to routine monitoring, we quickly established internal jugular venous access and parallel radial artery puncture. We also monitored the patient’s blood pressure and cardiac function using Vantage Flow. Subsequently, esketamine, propofol, etomidate, sufentanil, and rocuronium were chosen for the anaesthetic induction protocol, and the patient’s vital signs were observed to be stable after the induction.

**Fig. 1:**
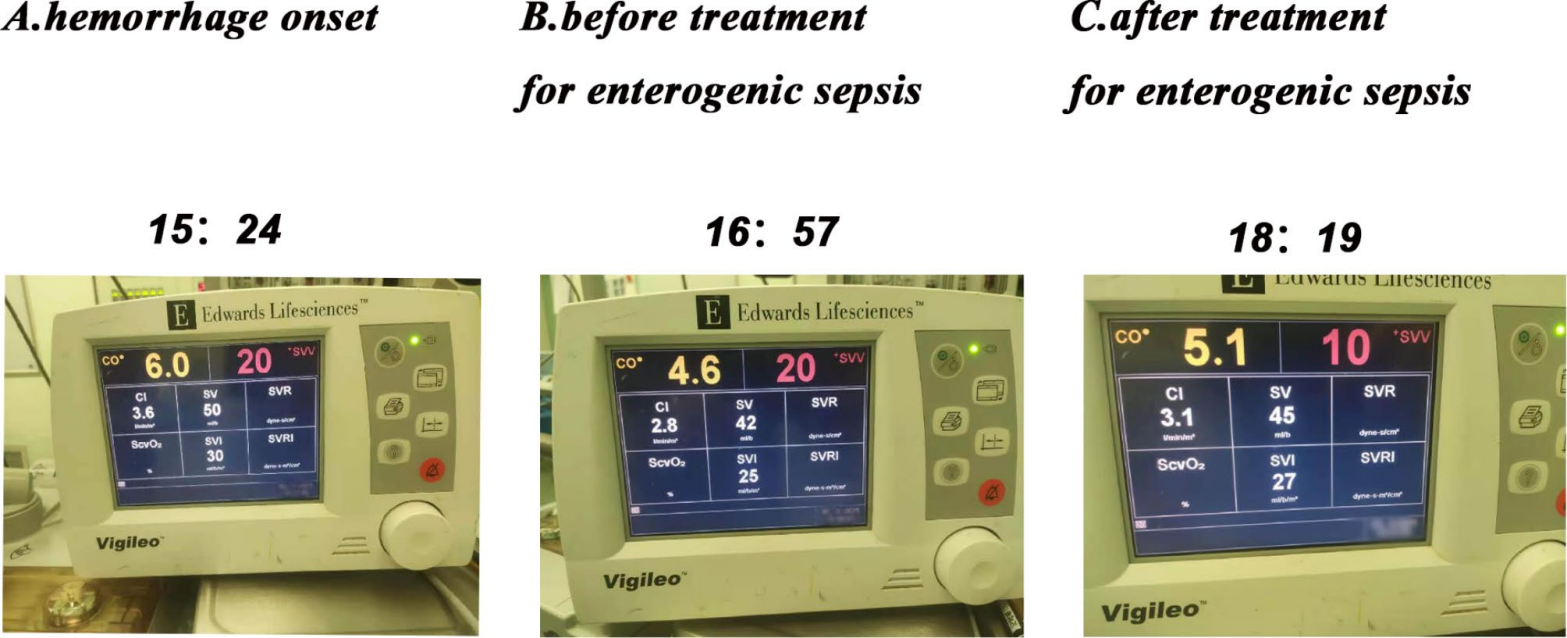
The variation of Vigileo’s parameters

At 14:50, a hemorrhage occurred during resection of the right half of the liver. In response, the patient’s blood pressure dropped rapidly to 70/52 mmHg, while the heart rate rose to 91 bpm. The rapid estimate of total bleeding volume of 2000 ml was made and an immediate intravenous infusion of 4 U of packed red blood cells and a rapid infusion of crystalloid was administered. Norepinephrine and meprobamate were used to maintain circulatory stability. In the following 4 h, the surgical wound continued to bleed (roughly 6000 ml), during which time we administered vasoactive drugs and goal-directed fluid therapy, including 4000 ml of lactated Ringer’s solution, 7 U of suspended red blood cells, and 900 ml of fresh frozen plasma. However, the patient’s hemodynamics neither improved significantly nor worsened. Meanwhile, systolic blood pressure fell to a minimum of 58 mmHg, a blood gas analysis suggested severe hyperlactatemia, and the patient’s urine output was significantly lower than before. All these evidence suggested that the patient remained in a state of severe water deprivation and tissue perfusion deficit during this time. We suspect that the severe hemorrhagic shock may have led to a pathophysiological alteration of sepsis with vasodilation, thus increasing endothelial permeability due to an intestinal barrier dysfunction, translocation of intestinal flora and endotoxins. The subsequent reduction in peripheral vascular resistance also supported our idea. We then accelerated the rate of rehydration and administered imipenem anti-infective therapy and corticosteroids supplementations. Following this, the patient’s circulation improved significantly, the systolic blood pressure stabilized at around 90 mmHg, and their urine output increased compared to before (The variation of Vigileo’s parameters is shown in Fig. 1). After the patient’s condition improved, we retained a bacteriological blood culture specimen.

Almost immediately following the above treatment, a second difficulty arose. At 20:30, as the surgeon was preparing to suture, the patient’s heart rate increased gradually, soon developing into supraventricular tachycardia (see supplemental material for details). To make matters worse, ventricular tachycardia with severe hypotension also occurred one minute later. The arterial blood gases measured at this time suggested the development of metabolic acidosis combined with hyperkalemia. We immediately corrected the arrhythmia with amiodarone and lidocaine, then administered glucose, insulin, sodium bicarbonate, and calcium gluconate via intravenous infusion. The assistant also prepared a defibrillator for use if necessary. Fortunately, the ventricular tachycardia soon returned to sinus and the blood pressure returned to normal. By 23:00, the operation was complete and the patient was safely admitted to the ICU. In total, 8600 ml of blood was lost, while a total of 19 U of suspended red blood cells, 1300 ml of fresh frozen plasma, 5500 ml of crystalloid. and 6500 ml of colloids were transfused.

**Fig. 2:**
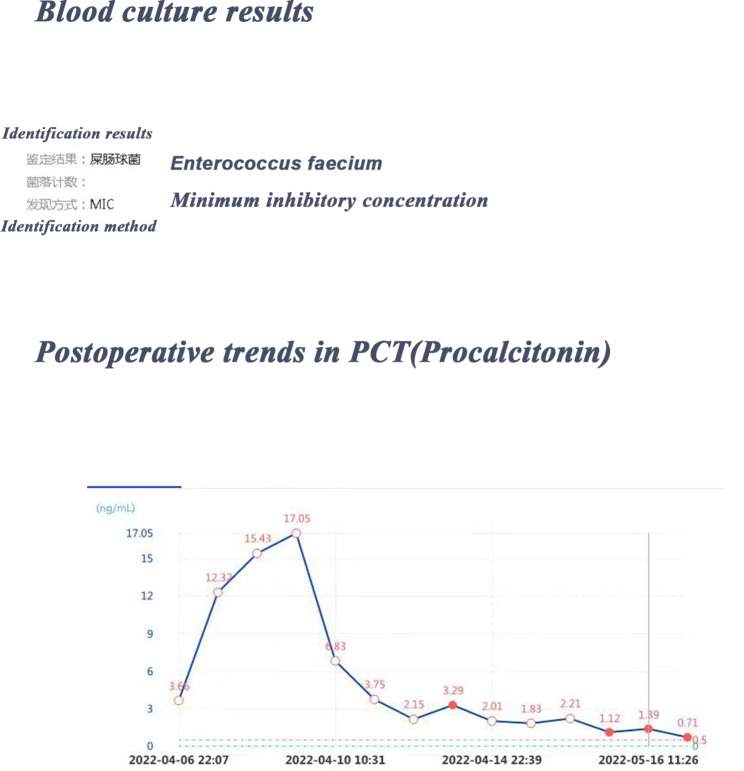
Postoperative trends in pct and blood culture results

Postoperatively, the patient showed a significant increase in inflammatory markers compared to that of the preoperative period as well as intraoperative blood culture specimen results for Enterococcus faecalis (See Fig. 2 for further details). The patient’s troponin, creatinine, liver function, and coagulation indexes were also elevated for a short period of time after surgery(see Table 1), suggesting combined heart, liver, kidney, and other multi-organ failure, which was considered a severe infection. Initially, anti-infective treatment with imipenem was given, and after the blood culture results returned, anti-infective treatment with vancomycin was switched. Polyene Phosphatidylcholine Capsules and glutathione were used for liver function protection. The patient was slightly irritable postoperatively and had mildly elevated blood ammonia. Therefore, we administered ornithine menthylate injection to improve blood ammonia metabolism and Semtex Transmetil to improve cholestasis. Postoperative coagulation indexes were significantly abnormal and platelets were significantly decreased, which was considered to be dilutional coagulopathy caused by massive fluid replacement after massive blood loss in a short period of time. We performed supplementation of coagulation factors and platelets. The rest of the treatment included sedation, analgesia, circulatory support, and nutritional support therapy.After these treatments,the patient was successfully transferred back to the general ward on the seventh post-operative day.

**Table 1:** Laboratory parameters in the preoperative and postoperative periods

	Pre	Surgery	POD1	POD2	POD3
Cardiac troponin I(ng/ml)		2.22	4.42	1.84	0.56
Prothrombin time(s)	10.6	46	16.8	15.4	16
Activated partial thromboplastin time(s)	27.9	> 170	33.7	36.3	38.9
D-dimer(mg/L)	0.23	9.01	7.67	6.43	13.64
Creatinine(ummol/L)	86	104	122	123	109
Aspartate aminotransferase(U/L)	61	1017	1778	2122	995
Alanine aminotransferase(U/L)	63		865	1244	850

Pre, preoperative period; Surgery, the day of surgery: POD, postoperative day.

## Discussion

In this case, the patient received preoperative antibiotic treatment for bile duct inflammation, showed no significant obstructive jaundice and the inflammatory indexes were within normal limits. Intraoperative blood culture suggested Enterococcus faecalis, and several blood cultures in the ICU still reported Gram-positive bacteria or Enterococcus faecalis. The postoperative anti-infection effect with vancomycin was good. Therefore, we considered that the patient had undergone emerging enterogenic sepsis. Cases of emerging sepsis due to intestinal barrier dysfunction and the displacement of intestinal flora during the perioperative period are quite rare. In the cases that do occur, most are commonly seen in combination with pre-existing combined sepsis or in gastrointestinal surgery. Because of its rarity, it is often easy to overlook the protection of the intestinal barrier in the perioperative period. However, its impact on management is still significant.

The identification and diagnosis of perioperative sepsis of intestinal origin is uniquely relegated to the likes of wards and ICUs. This is because while in surgery, the patient is always under anesthetic sedation. While they are in this state, we can only assess the patient’s condition using the parameters of the monitoring instruments and, as a result, many clinical manifestations and complaints can be masked. For example, the patient’s state of consciousness cannot be properly assessed. This is also due to the fact that the patient in this case was experiencing both hemorrhagic shock and sepsis, both of which cause similar effects such as hypotension, metabolic acidosis, oliguria, and so on. Finally, the diagnosis of enterogenic sepsis in general wards relies on microbiological tests, including blood cultures, fecal cultures, or cultures of nasogastric aspirates [4]. However, as the results of the tests are often not immediately available to us during the procedure, capturing the enteric origin on the basis of hemorrhagic shock is a challenge.

The patient had a pre-operative base of cirrhosis. This also likely effected the outcomes, as current studies show that hemorrhagic shock, intestinal obstruction, cirrhosis, obstructive jaundice, and acute pancreatitis are all risk factors for intestinal flora and endotoxin translocation [4, 5]. During the course of the surgery, the patient experienced intraoperative hemorrhagic shock and we believe the patient was at high risk of intestinal flora translocation. Throughout the second half of the procedure, even after aggressive fluid resuscitation and the administration of vasoactive drugs, the patient continued to demonstrate paradoxical water deficit, inadequate tissue perfusion and low peripheral vascular resistance. In combination with the patient’s history and clinical presentation, we had good reason to suspect the possibility of enterogenic sepsis. The positive follow-up feedback also supports our continued treatment of enterogenic sepsis.

To facilitate the rapid identification of sepsis, particularly sepsis of intestinal origin, there are various screening tools that are now widely used in clinical practice. In addition to traditional screening tools such as Systemic Inflammatory Response Syndrome (SIRS), Sequential Organ Failure Assessment (SOFA) criteria and National Early Warning Score (NEWS), there have been several previous studies that have attempted to establish a system for identifying septic shock in patients with intestinal infections. For example, Peiling Chen et al. established a similar early warning scoring system for infection shock in patients with gastrointestinal perforation. It assessed items such as heart rate, lactate, calcitoninogen, C-reactive protein and its Grass score [6]. In their study, Islam M.M. et al. applied mechanistic learning to the prediction of sepsis with better sensitivity and specificity than traditional screening tools [7]. However, most current screening tools cannot be applied during anesthetic management due to the anesthetic state of the patient during surgery and the time it often takes to return test results. Further research should be conducted in the future to develop screening tools and identification systems that effectively offer early warnings of perioperative enterogenic sepsis.

Cardiac arrhythmias in patients with sepsis are often a challenge for anesthetists in the perioperative period. Amiodarone is now the first choice for the correction of arrhythmias due to its low cardiac suppression side effects [8–11]. Notably, previous studies have shown that the success rate of electrical cardioversion is significantly higher in septic patients who have used amiodarone compared to those who have not used arrhythmia medication [12].

## Conclusion

Although rare, the occurrence of intrahepatic hemorrhaging combined with emerging enterogenic sepsis during a hepatectomy poses a major challenge and can be fatal. Familiarity with the risk factors and pathophysiological alterations of enterogenic sepsis is a prerequisite for early recognition and sound clinical decision making. In the face of such arrhythmias that occur in patients with sepsis, amiodarone is considered to be a good option.

## Electronic supplementary material

Below is the link to the electronic supplementary material.

Supplementary Material 1

Supplementary Material 2

## Data Availability

All data related to this case report are contained within the manuscript.
